# Emergency paediatric medicine consultation—a practical guide to a consultation with refugee and asylum-seeking children within the paediatric emergency department

**DOI:** 10.1007/s00431-023-05067-0

**Published:** 2023-07-21

**Authors:** Jaya Chawla, Nour Houbby, Sarah Boutros, Sarah Davies, Ella Farina, Charles G. Stewart, Osama Munajjed

**Affiliations:** 1 London North West Healthcare Trust, London, UK; 2 Barts Health NHS Trust, London, UK; 3 Guy’s and St Thomas’ Foundation Trust, London, UK; 4 Chelsea and Westminster Foundation Trust, London, UK; 5 Imperial College London, London, UK

**Keywords:** Child health, Communicable diseases, Global health, Mental health, Paediatric emergency medicine

## Abstract

There are increasing numbers of refugee and asylum-seeking children entering the UK annually who face significant barriers to accessing healthcare services. Clinicians working in the emergency department should have an awareness of the journeys children may have taken and the barriers they face in accessing care and have a holistic approach to care provision. We conducted a narrative literature review and used experiential knowledge of paediatricians working in the Paediatric Emergency Department to formulate a step-by-step screening tool. We have formulated a step-by-step screening tool, CCHILDS (Communication, Communicable diseases, Health—physical and mental, Immunisation, Look after (safeguarding), Deficiencies, Sexual health) which can be used by healthcare professionals in the emergency department.

*Conclusion:* Due to increasing numbers of refugee and asylum-seeking children, it is important that every point of contact with healthcare professionals is an impactful one on their health, well-being and development. Future work would include validation of our tool.

**Table:** 

**What is Known:**
•*The number of refugees globally are rapidly increasing, leading to an increase in the number of presentations to the PED. These patients are often medically complex and may have unique and sometimes unexpected presentations that could be attributed to by their past. There are a multitude of resources available outlining guidance on the assessment and management of refugee children.*
**What is New:**
•*This review aims to succinctly summarise the guidance surrounding the assessment of refugee children presenting to the PED and ensure that healthcare professionals are aware of the pertinent information regarding this cohort. It introduces the CCHILDS assessment tool which has been formulated through a narrative review of the literature and acts as a mnemonic to aid professionals in their assessment of refugee children in the PED.*

## Introduction

The number of refugees worldwide has increased considerably due to ongoing wars, national instability, climate change, political persecution and food and economic insecurity [1]. The countries of origin of refugees and asylum seekers have varied over recent years, depending on factors including political crises, war, conflicts and climate emergencies [2]. For example, the recent turmoil in Ukraine has led to the evacuation of thousands of refugees and asylum seekers [2]. Within the UK, as of September 2022, the top five countries for asylum applications included Iran (6002), Eritrea (4412), Albania (4010), Iraq (3042) and Syria (2303) [1]. By the end of 2022, almost 40% of refugees globally are children and adolescents under the age of 18 [3]. In 2018, 74% of UASC were 16–17 years old, 21% were 14–15, whilst 2% were under 14 with a total of 89% of applicants being male [4, 5].

Refugee and asylum-seeking children (Table 1) face significant risks to their health and therefore may have complex health needs [6]. Therefore, establishing holistic care for refugee and asylum-seeking children can have widespread positive implications and may shape their transition into a new country.

**Table 1:** Terminology involved and what this means for the child in question

**Terminology**	**Definition**	**Implications**
Refugee	Someone who is unable or unwilling to return to their country of origin owing to a fear of being persecuted for reasons of race, religion, nationality, membership of a particular social group or political opinion	Once the government agrees that a person seeking asylum is successful in their application, they will be issued refugee status and documentation. In the UK, they are given 5 years’ leave to remain as a refugee, after which, they must apply for further leave
Person seeking asylum	A person who has left their country of origin and formally applied for asylum in another country but whose application has not yet been concluded	It must be considered that there is a chance these applications could be rejected, and the person seeking asylum will be required to return home. At this stage, the person cannot work pending a decision from the government. If the appeal is rejected, there may be grounds to consider appeal
Refused asylum seeker	A person whose asylum application has been unsuccessful and who has no other claims for protection awaiting a decision	Some refused asylum seekers return home voluntarily; others are forcibly returned. For many, it is not safe or practical to return
Unaccompanied asylum-seeking children (UASC)	A child or young person seeking asylum in the UK who: • is under 18 years of age when the claim is submitted • is claiming in their own right • is separated from both parents and is not being cared for by an adult who has responsibility to do so	Whilst these children have their claim processed, they are looked after by a local authority. These children should have an initial health assessment, most commonly by a community paediatrician

Within their country of origin, war, political conflict and human rights violations may have resulted in a lack of adequate healthcare which can lead to undiagnosed or untreated chronic conditions, an increased risk of vaccine-preventable communicable diseases, nutritional deficiencies, STIs and mental health issues [7]. Moreover, the unsafe journey can lead to serious health complications such as hypothermia, infections, malnutrition, dehydration and traumatic injuries [8].

There is also a high prevalence of mental health issues across refugee and asylum-seeking children including post-traumatic stress disorder, depression and anxiety disorders from either witnessing or suffering traumatic experiences such as sexual abuse and violence, isolation and the burden of leaving family in their home country [9].

A notable study by Nijman et al. [10] found that there is a necessity for improved guidance, educational tools and a platform for shared learning on managing refugee children in emergency care. There are multiple obstacles faced to accessing healthcare including language barriers, financial limitations and a lack of trust in the system. Furthermore, numerous system obstacles are faced which include frequent relocations, administrative issues and a fear of health information being shared with the Home Office for refused asylum seekers. This paper aims to provide a concise summary of what paediatric emergency medicine healthcare professionals should know regarding the journey taken by refugee and asylum-seeking children and the barriers to accessing healthcare and provide a framework for assessing patients.

## Key pre-consultation considerations

An awareness of the refugee or asylum-seeking child’s background and journey to the UK is necessary to provide effective, holistic care within the PED. We outline below key considerations. Please note it is not expected you would gather all the below information during a consultation. Rather, these are factors to consider when assessing children in the PED, ensuring culturally sensitive and trauma-informed practice.

- Background
  - Life context of the child and family: religion, gender, ethnic origin and individual circumstances. They may have been exposed to several traumatic events during childhood which can predispose to the development of mental health conditions, which are later explored [11].
  - Reasons for migration and profile of the country of origin: main challenges, disease prevalence, impact of war and political crisis.
  - The healthcare access in the country of origin: what is the health infrastructure like (i.e. is healthcare free), what preventative care is in place?
  - Timeline of medical care from antenatal care to current presentation: likelihood of adequate antenatal screening (e.g. HIV, hepatitis B, foetal alcohol syndrome), access to postnatal screening routinely conducted in the UK (e.g. Guthrie test, new-born baby check and hearing screen) and vaccination schedules.
  - Refugee and asylum-seeking children may be malnourished and have micronutrient deficiencies.

- During the journey
  - Consider route and mode of travel; however, be aware that many patients may be reluctant to share details.
  - Those travelling alone, especially women and children, are at a higher risk of sexual assault during their travel [12]. Hence, consider possible psychological distress, risk factors for blood-borne infections, STIs, urogenital damage and pregnancy.
  - Depending on the time spent in camps or other cramped conditions, there may be an increased risk of contracting TB and other infectious diseases.

- Arrival to the UK
  - Refugee and asylum-seeking children and young people may be subject to frequent changes to their accommodation which can lead to disjointed healthcare.
  - There may be difficulties faced in accessing healthcare due to a lack of money and transportation.
  - Complex and lengthy immigration processes, frequent relocations, difficulties in accessing healthcare, education and other sources of support can all contribute to increased emotional distress.

## Barriers to accessing healthcare

For those who settle in the UK, there are many barriers to accessing health and social care. There may be a lack of knowledge of their rights and entitlement within the UK health system; administrative barriers; and cultural and language barriers [12]. There may also be concerns about facing discrimination from healthcare professionals.

### Rights and entitlement within the UK health system

Many refugee and asylum-seeking children are unaware of their rights and entitlement within the UK health system [13]. Although there is a lack of resources available to PED healthcare professionals regarding refugee and asylum-seeking children, efforts should be made to provide coherent information on the entitlement and rights to services at all points of contact. This empowers patients so that they can better advocate for themselves. The Refugee Council [14] provides a factsheet in multiple languages containing information on healthcare eligibility and access for people seeking asylum in the UK. Crucially, in England, there is no minimum time that a patient must reside in the UK to be eligible to receive NHS primary medical care services [13].

Despite those rights, there are well-documented barriers to accessing healthcare for this population. Administrative barriers, such as needing ID documents and proof of address to register at most GP surgeries, cause delays and denials of GP registration. It is vital to be aware that everyone should be able to access primary healthcare services without any documentation [15, 16]. NHS charging regulations, which exempt asylum seekers and refugees from charging, have been found to be at times inappropriately applied in this population leading to delays and denials of care [17]. Language barriers and the lack of readily available interpreters in all healthcare settings can cause further obstacles. Financial difficulties can also make it hard to get to appointments, particularly in secondary care. Frequent relocations in the UK by the Home Office can cause disjointed care provision. Finally, there may be well-founded reasons to distrust healthcare services, particularly as healthcare charging regulations can lead to sharing of information between healthcare settings and the Home Office, particularly for refused asylum seekers who are at risk of deportation [14].

The barriers described above can mean that asylum seekers and refugees are less likely to access healthcare in Emergency Departments.

## PED consultation

**Fig. 1:**
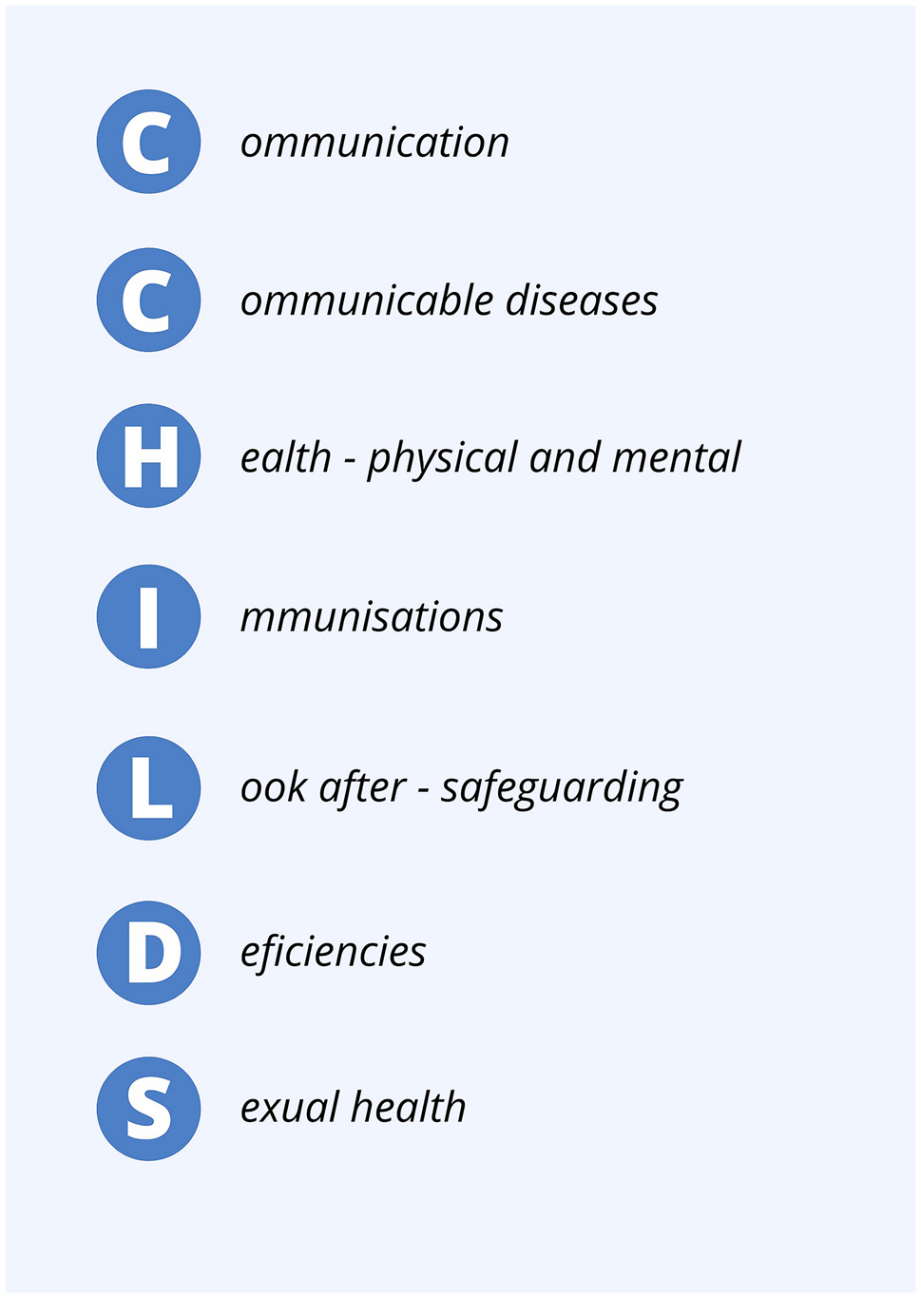
CCHILDS assessment illustration

A structured, comprehensive history should be taken with efforts to adopt a holistic approach towards the child and family. We have established a step-by-step screening tool (Fig. 1) and created the acronym CCHILDS to aid in the assessment of refugee and asylum-seeking children. This tool is not currently validated; however, it has been formulated to aid in the assessment of refugee and asylum-seeking children presenting to PED.

## Methodology

The CCHILDS assessment tool (Fig. 1) was formulated through a narrative assessment of the literature on child and refugee health. A literature search of PUBMED, OVID Medline and OVID Embase was conducted to synthesise key evidence on refugee and asylum-seeking children health considerations. This was supplemented with a detailed Google search and reference searching to generate areas of focus in the assessment of the paediatric refugee or asylum-seeking children. The seven areas of focus we identified were the elimination of language barriers, screening for infectious diseases, physical health examination, immunisations, screening for safeguarding concerns, nutrition screening and sexual health. These areas were then streamlined into a mnemonic for ease of memory.

## CCHILDS step-by-step acute healthcare screen

### Communication

- Use a professional, third-party interpreter, if required, to create a safe and trusting atmosphere [18]. If there are difficulties in ascertaining an appropriate interpreter immediately, mobile phone translation applications (e.g. Care to Translate) may be used to aid the consultation but used with caution and consideration as these applications are not validated and must not replace a professional interpreter [19].
  - Family members should not be used as interpreters as they are unlikely to provide neutral, unbiased opinions and there may be potential for exploitation.
- Use trauma-informed care approaches in all encounters as many will have experienced traumatic events that will affect their health, mental health and all aspects of their lives. Trauma-informed care principles include safety, trust, choice, collaboration, empowerment and cultural consideration [20].

### Communicable and parasitic diseases

Screening for infectious diseases should be guided by clinical presentation and risk. Screening is also recommended for asymptomatic communicable diseases since refugees and asylum seekers are at increased risk.

**Table 2:** Four key resources regarding refugee health for ED healthcare professionals

**Migrant A to Z guide, Gov.uk **[22]	Comprehensive list of all countries with individualised health needs. It is a useful tool to identify prevalent communicable diseases in specific countries
**RCPCH **[43]	Key information for paediatricians with advice on promoting refugee child health
**Doctors of the World **[35]	Provides posters in different languages with information for migrants on their access to healthcare. Provides healthcare and access to clinics to all migrants regardless of refugee status and are contactable via their website
**World Health Organisation **[44]	Up-to-date information regarding child refugee health
**Centre for Disease Control and Prevention **[45]	Vast resource for country-by-country and region-by-region data and advice particularly on communicable disease

- Asymptomatic screening includes (but is not exclusive to) tuberculosis (TB), hepatitis, HIV, sexually transmitted infections and parasitic infections [21].
- Clinical evaluation of the most prevalent diseases from the country of origin should be considered when presented with symptoms. This can be aided by the Migrant A-Z guide [22] from Public Health England (Table 2) and it is advised to make appropriate referrals to infectious diseases.
- Children or young people presenting from countries with an incidence of TB of, or greater than, 40/100,000 should be referred to paediatric TB or infectious diseases services for assessment [21]. Latent TB screening may also be conducted in PED.
- If clinically indicated, it may also be relevant to screen for tropical parasitic diseases including bilharziasis, anguillulose and malaria.
- Young people at risk of STIs and blood-borne viruses may be asymptomatic. Routinely offer to screen for chlamydia, gonorrhoea, syphilis, hepatitis B and C and HIV to reduce the risk of stigmatisation from target screening [18]. This could be followed up by the GP or by referral to appropriate secondary care services (e.g. paediatrics infectious diseases) dependent on the presentation.
- For concerns about child sexual abuse, this needs to be escalated to seniors immediately and the local guidelines should be followed. This will include referral to social care and specialist child sexual abuse services.

Where appropriate, discussions around the importance of communicable disease testing should occur with the child/adolescent away from the bedside.

### Health—physical and mental

- Mental health:
  - In the acute setting, it is important to be aware that exploring previous traumatic experiences and psychological trauma is not routinely recommended; they are important considerations but should not be specifically questioned during the consultation unless the patient is presenting with an acute mental health condition. It is likely to cause additional distress on the child with no guaranteed social work or psychology follow-up.
  - If mental health is an acute issue on presentation, it is recommended to address this and refer to CAMHS. Third sector services (e.g. Refugee Council) can offer further support [14].
  - There are a multitude of considerations that may impact the child and contribute towards their presentation. These are summarised in specific guidance for paediatricians assessing refugee and asylum-seeking children, available on the RCPCH website [21].

- Physical health:
  - Take a comprehensive history and conduct a thorough physical examination as would be done for any patient but with a particular focus on the following [23, 24]:
    - Malnutrition
    - Poorly managed chronic health conditions
    - Dental health—discuss any dental symptoms and signpost to attend NHS dental practice as soon as possible even if asymptomatic. Educate patients and families around the importance of oral hygiene and lifestyle choices such as smoking and alcohol which are associated with adverse oral health outcomes.
    - Access to vaccinations
    - Trauma and injury
    - Infections
    - Development
    - Lack of health screening and health promotion
    - Baseline bloods, nutrition screen, infectious disease screen

### Immunisations

- Establish vaccination history and provide information on the UK immunisation schedule [25].
- If the vaccine history is uncertain, opportunistically vaccinate if possible and provide information on GP registration, recommending a full course of catch-up immunisations.

### Look after—safeguarding

- Immediate referral to local authority for all children presenting alone.
- If there are indications of possible trafficking, sexual assault, female genital cutting, mental health issues or other safeguarding concerns, discuss with seniors and make appropriate referrals according to Local Hospital Guidelines. It is vital that these topics are discussed with a non-stigmatising approach with care taken regarding the language used (e.g. ‘mutilation’ may be found offensive with ‘cutting’ and ‘circumcision’ preferred internationally).
- Exposure to violence, rape and/or other traumas should be explored sensitively. Not all young people are able to disclose on first assessment if they have been the victim of assault, and this will need careful inquiry. However, be aware that this may not always be relevant or appropriate in the PED setting with no formal follow-up. It is also important to differentiate between historical child protection concerns and current acute risk.
- Be aware of cultural child protection concerns including the risk of underage/forced marriage and exposure to child labour which may be more prevalent in certain ethnic cohorts [26].
- Safeguarding concerns may arise from an unsafe environment for the child in temporary accommodation or due to neglect or physical abuse [18].
- For a young person experiencing bullying, racism and other forms of marginalisation, there is increased vulnerability to exploitation.

### Deficiencies

- Refugee and asylum-seeking children are at a high risk of malnutrition and micronutrient deficiencies [27].
- Food insecurity remains a common concern following resettlement in developed countries. Targeted, culturally appropriate parental education resources and interventions may help mitigate this following resettlement, although may not be clinically relevant in PED unless directly corresponding to the patient’s presentation [28].
- Consider screening for anaemia, micronutrient and vitamin deficiencies when requesting blood tests. Be aware of micronutrient deficiencies such as vitamin B complex, vitamin A and zinc with night blindness being a common sign of vitamin A deficiency. It is also important to note that conditions such as vitamin D deficiency are more likely to lead to rickets in this cohort and hence, it is advisable to consider bowing of legs, widened wrist epiphyses and a soft skull in infants.

### Sexual health

- Signpost or offer to arrange referral for adolescent or ‘at-risk’ patients to local sexual health services. Test for HIV and hepatitis B and C in at-risk groups with consent.
- Considerations must also be made regarding the adolescent’s reproductive health. Limited education, availability of preventative vaccines and contraception use may contribute to high-risk sexual behaviours and will increase the risk of unplanned pregnancies and STIs in this cohort. Be aware of cultural stigma regarding sexual and reproductive health and the impact this may have on the consultation [29].
- If there is a concern about possible child sexual abuse (CSA), refer to social care and refer to local CSA services.

We appreciate that this is a vast amount to cover in a stretched PED with only 15 min per consultation. Although all the points are unlikely to be covered, it is important for the clinician to be aware of guidelines and the main considerations so that their consultation can be appropriately focussed on managing the specific needs of a refugee or asylum-seeking child where possible. In particular, the main take-away point for clinicians is to use a professional interpreter wherever there is limited English proficiency and not to use family or friends unless it is a life-threatening situation. Advice regarding the use of interpreters can be found on the GOV.UK website [30] and also on the BMA [31]. Furthermore, Migrants Organise [32] have issued a “*Good practice guide to interpreting*” which is useful for both clinicians and patients alike and available in a multitude of languages.

## Discharge from PED: things to consider

Upon discharge from the PED, there are important considerations universal to all paediatric refugee and asylum-seeking children.

1. Explain how the NHS works and entitlement to care
  - Refugees and asylum seekers are eligible to receive all NHS care free of charge [33].
  - Explain the range of NHS services available including emergency, primary and secondary care [34].
  - Social care depends on the local authority guidelines and appointment of a social worker is conducted on a case-by-case basis.
  - All children in the process of claiming asylum or have been granted refugee status have access to education. UASC will have an allocated social worker.
  - Provide discharge letters to ensure they have all documentation should the patient be transferred elsewhere. This may be subject to decision by the Home Office.
2. Consider acute referrals if appropriate, for example to secondary care, CAMHS or social services as many children will not be registered with GP.
  - Social workers can support with GP registrations and can make CAMHS referrals if needed.
  - Consider arranging secondary care paediatric follow-up if needed whilst awaiting GP registration.
3. Facilitate registration with a GP
  - Explain that registration with a GP is free and encourage this. There are leaflets by Doctors of the World [35] (Table 2) that can be printed out for the patient to take to the GP.
  - Identify local GP via the ‘Find a GP’ NHS site [36] or register using a GMS1 form [37]. Print their discharge summary and a written note to the GP explaining the patient’s current situation and why they need registration.
    (i) Alternatively, call ‘Doctors of the World’ and book into one of their free clinics [38].
4. Be mindful of financial limitations
  - Consider prescribing over-the-counter medications, being mindful of monetary limitations commonly faced by refugee and asylum-seeking children (the UK government currently issues £40.85 per week to each refugee to help pay towards food, clothing, toiletries and all other basic necessities) [39]. Prescriptions for children are free of charge and adults would need a HC2 certification for evidence of exemption [40].
5. Social and education considerations
  - Aim to signpost children and their families to school registration. Refugee and asylum-seeking children often suffer from interrupted education due to limited resources, living in poverty and migration which affects their access to education [41, 42].

## Limitations and scope for future work

There are several limitations of this review. Firstly, this is a narrative review in practice aimed at providing a practical approach to the assessment of refugee and asylum-seeking children and therefore may not have captured evidence that may have been synthesised in a systematic review. In addition, the CCHILDS tool has been formulated using the literature on refugee and asylum-seeking children; however, this tool has not been validated.

Future work could look at validating the CCHILDS tool and evaluating its use in PED settings.

## Summary

Facilitating quality healthcare to refugee and asylum-seeking children is key to aiding health, well-being and development. With increasing numbers of refugee children, the opportunities for positive impact at each point of contact is essential. We recommend adopting the CCHILDS approach for the clinical evaluation of paediatric refugees and asylum seekers who present to the PED.

## Abbreviations

CAMHS: Child and Adolescent Mental Health Services
CCHILDS: Communication, Communicable diseases, Health-physical and mental, Immunisations, Look after-safeguarding, Deficiencies, Sexual health
GP: General Practice
PED: Paediatric Emergency Department
RCPCH: The Royal College of Paediatrics and Child Health
STIs: Sexually transmitted infections
TB: Tuberculosis
UASC: Unaccompanied asylum-seeking children
